# Pilot Study on MAGE-C2 as a Potential Biomarker for Triple-Negative Breast Cancer

**DOI:** 10.1155/2016/2325987

**Published:** 2016-10-24

**Authors:** Qian Zhao, Wen-ting Xu, Tuluhong Shalieer

**Affiliations:** Department of Breast Surgery, The Affiliated Tumor Hospital of Xinjiang Medical University, Urumqi, Xinjiang 830011, China

## Abstract

*Objective*. In the current study, we measured the expression status of melanoma antigen gene c2 (MAGE-C2) in triple-negative breast cancer (TNBC) and analyzed its prognostic with the clinical pathological features of patients with TNBC.* Methods*. The expressions statuses of MAGE-C2 were detected in TNBC tissues and paracarcinoma tissues by immunohistochemistry, reverse transcription-polymerase chain reaction (RT-PCR), and western blotting. Then, we investigated the relationship of MAGE-C2 expression status and clinicopathological parameters of TNBC patients by the chi-squared test. Finally, we discussed the relations of MAGE-C2 expression state and prognosis of patients with TNBC by Kaplan-Meier method and Cox proportional hazards model.* Results*. High MAGE-C2 expression was found in 38.18% (42/110) of TNBC tissues. In adjacent tissues it was 9.09% (10/110). High MAGE-C2 expression in TNBC patients was closely associated with lymph node status, tumor node metastasis (TNM) stage, and lymphovascular invasion (*P* < 0.001). TNBC patients with high MAGE-C2 expression had significantly shorter survival time than low expression patients. We also found that age, lymph node status, TNM stage, lymphovascular invasion, and MAGE-C2 expression status were closely associated with overall survival of TNBC patients (*P* < 0.05).* Conclusion*. High MAGE-C2 expression may serve as an independent prognostic factor for TNBC patients.

## 1. Introduction

The morbidity of breast cancer ranks the highest among female malignancies worldwide, and it is approximately 42.55/100,000 in China [1, 2]. Breast cancer has become the main cause of death of Chinese women, with an annual increase of 3% in recent years [2]. Breast cancer can be divided into the luminal A subtype, luminal B subtype, human epidermal growth factor receptor 2- (Her-2-) positive subtype, and triple-negative breast cancer (TNBC) subtype based on the molecular types. TNBC, which is characterized by a poor prognosis, high recurrence and metastatic rates, and high mortality, accounts for about 10% to 16% of all breast cancer cases [3]. Triple-negative breast cancer (TNBC) is a high aggressive subtype, which is not sensitive to endocrine therapy, or targeted therapy, and the main treatment options are limited to surgery, radiotherapy, or chemotherapy. Therefore, there is an urgent need to search for the key targets of TNBC treatment, which is the focus of this study.

The melanoma antigen gene (MAGE) family is a group of cancer testis antigens, which are isolated from melanoma cells [4]. MAGE antigens are not expressed in normal tissues other than the testis. However, moderate to high MAGE expression can be seen in some malignant tumors. MAGE-C2 can be degraded into nonapeptide or decapeptide in the cytoplasm of tumor cells and combine with HLA molecules in the cells as the epitope, which is subsequently presented on the cell membrane, inducing the body's immune system to produce relevant antibodies [5]. It has been verified that abnormal expression of MAGE-C2 can be found in adenocarcinomas of lung cancer, liver cancer, laryngocarcinoma, prostate cancer, and non-TNBC [6–9]. However, there is little research on the relationship between MAGE-C2 and TNBC. We speculate that delayed tumor growth, proliferation, infiltration, and metastasis were attainable by blocking melanoma antigen gene c2 (MAGE-C2) related signal transduction pathways. In the current study, the expression status of MAGE-C2 mRNA and protein in TNBC is measured by us, and we analyzed their relations with the clinical pathological features of patients with TNBC. We further demonstrate that MAGE-C2 is a novel biomarker of TNBC, which play critically important role in TNBC development.

## 2. Materials and Methods

### 2.1. Patients and Tumor Specimens

A total of 110 samples of tumor and paracarcinoma tissues from TNBC patients who underwent surgical resection in the Affiliated Tumor Hospital of Xinjiang Medical University from May 2008 to August 2010 were collected. The tumor and adjacent tissues were fixed in paraffin. All patients were pathologically diagnosed and had complete clinical data. The patient age range was 22 to 79 years, with a median of 58.3 ± 7.5 years. None of the 110 TNBC patients received chemotherapy, radiotherapy, or biotherapy preoperatively. The study was approved by the ethics committee of the Affiliated Tumor Hospital of Xinjiang Medical University. All patients or their family signed an informed consent.

### 2.2. Immunohistochemistry

The paraffin samples were cut into 4 *μ*m thick slices, which were then placed in a 60°C oven overnight. Conventional dewaxing was conducted until hydration, and the samples were incubated with freshly prepared 3% hydrogen peroxide in deionized water to seal the endogenous peroxidase at room temperature for 10 min. A high-pressure hotfix was performed with 0.01 mol/L citrate buffer solution (pH = 6.0), followed by staining according to the instructions for the immunohistochemical kit. Rabbit polyclonal anti-MAGE-C2 antibody (1 : 200; Sigma-Aldrich, Shanghai, China) was added dropwise, and the samples were incubated overnight in a refrigerator at 4°C. The samples were then washed with PBS, and the secondary antibody was added dropwise, followed by incubation at 37°C for 10 min. The samples were washed with PBS three times, and streptavidin-horseradish peroxidase was added dropwise, followed by incubation at 37°C for 10 min. The samples were washed with PBS three times, the color was developed with DAB while controlling the developing degree under a microscope, and staining was terminated with distilled water.

The samples underwent a pathological evaluation using a single-blind method (the pathologists were blinded to the clinical data), and five medium views were selected randomly (magnification of 200x), and in each view 200 tumor cells were counted, for a total of 1,000 cells. MAGE-C2 protein staining in tumor and normal tissues was scored according to the following standards: staining intensity was classified as 0 (lack of staining), 1 (mild staining), 2 (moderate staining), or 3 (strong staining); the percentage of staining was designated 0–100%. For each section, the semiquantitative score was calculated by multiplying these two values. We defined a MAGE-C2 staining intensity of 0 points or 1 point as low expression and that of 2 or 3 points as high expression.

### 2.3. Reverse Transcription-Polymerase Chain Reaction (RT-PCR)

The RNA in the tissues was extracted using the Trizol method, and the RNA was then reverse-translated into cDNA and used as a template for PCR amplification. The above-mentioned procedure was performed in strict accordance with the kit's instructions. The PCR reaction conditions were as follows: 35 cycles of predegradation at 94°C for 2 min, degradation at 94°C for 30 s, annealing for 30 s, and extension at 72°C for 1 min, followed by 72°C for 10 min. Then, 3 *μ*L aliquots of the PCR reaction products were subjected to 2% agarose gel electrophoresis, and the gray values of the mRNA bands were analyzed using Quantity One software. The relative expression level of MAGE-C2 mRNA was expressed as the ratio of the MAGE-C2 mRNA band's gray value to that of the internal reference MAGE-C2 mRNA. The forward primer for MAGE-C2 was 5′-AAAGTCAGCACAGCAGAGGAG-3′, and the reverse primer was 5′-TCTTCAGGAGCAGCAGGTAAA-3′.

### 2.4. Western Blotting

A 100 mg sample of tissue was placed in 500 *μ*L protein lysis buffer and ground in a homogenizer. The sample was then pyrolyzed on ice for 30 min and then centrifuged (12,000 r/min) to collect the supernatant, and the protein concentration was determined using the BCA method. The protein samples were loaded on a 10% polyacrylamide gel for electrophoretic separation and sealed for 1 hr with 5% BSA. Then, rabbit polyclonal anti-MAGE-C2 antibody (1 : 1,000) and GAPDH mouse anti-human monoclonal antibody (1 : 1,000; Santa Cruz Biotechnologies, Santa Cruz, CA, USA) were added. After incubation at 4°C overnight, the secondary antibody (1 : 2,000; Bio-Rad, Hercules, CA, USA) was added followed by incubation at room temperature for 1 hr, after which the chemiluminescence reagents were added. The relative content of MAGE-C2 is presented as the ratio of the MAGE-C2/GAPDH gray values, and the gray values were analyzed by Quantity One software (Bio-Rad Laboratories, Philadelphia, PA, USA).

### 2.5. Statistical Analysis

All statistical analyses were performed using SPSS 19.0 (IBM, Armonk, NY, USA). The count data of the two groups were compared by the chi-squared test. In the present study, we selected the disease-free survival (DFS) and overall survival (OS) as main outcomes of patients with TNBC. The Kaplan-Meier method was used for survival analysis, and the log rank test was used to compare patient survival between the two MAGE-C2 expression groups. To further analyze the survival data, the Cox proportional hazards model was used for joint effect analysis of each covariate. All statistical tests were two-tailed, and *P* < 0.05 was considered significant.

## 3. Results

### 3.1. MAGE-C2 Expression in TNBC

**Figure 1:**
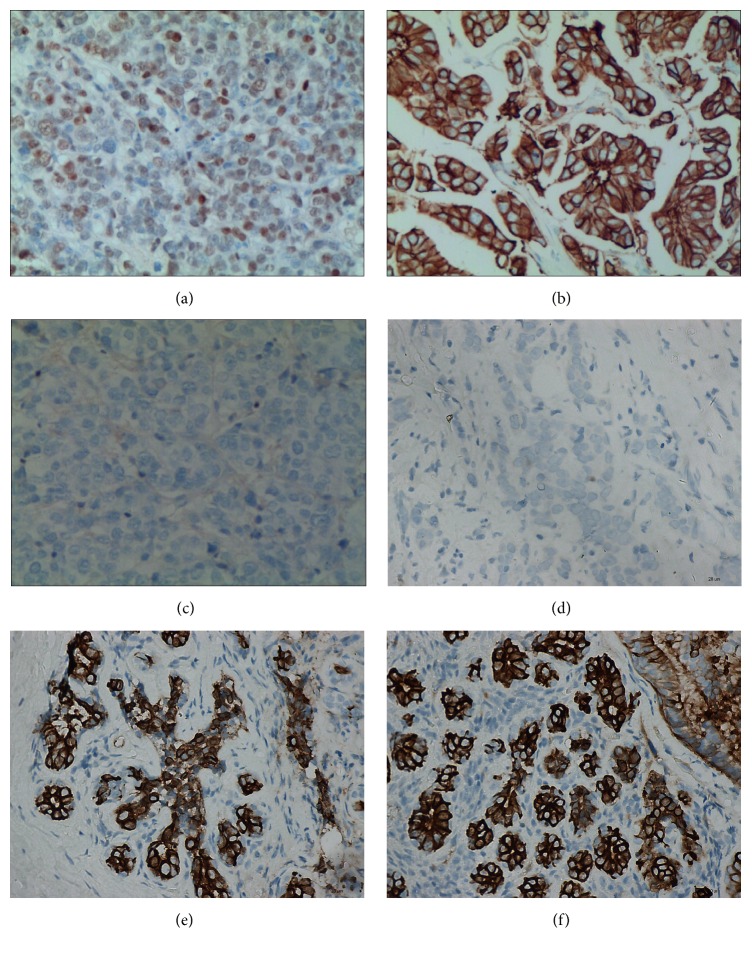
The MAGE-C2 protein expression in TNBC tissues and adjacent tissues detected by immunohistochemistry. (a and b) high MAGE-C2 protein expression in TNBC tissues; (c and d) low MAGE-C2 protein expression in TNBC tissues; (e and f) MAGE-C2 protein expression in adjacent tissues. Note: TNBC: triple-negative breast cancer.

**Figure 2:**
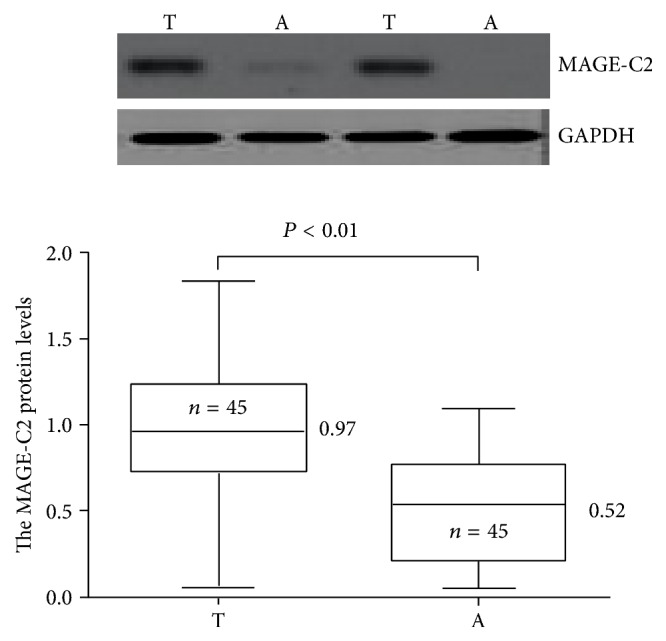
The MAGE-C2 protein was detected by western blotting. Note: A: adjacent tissues; T: triple-negative breast cancer tissues.

**Figure 3:**
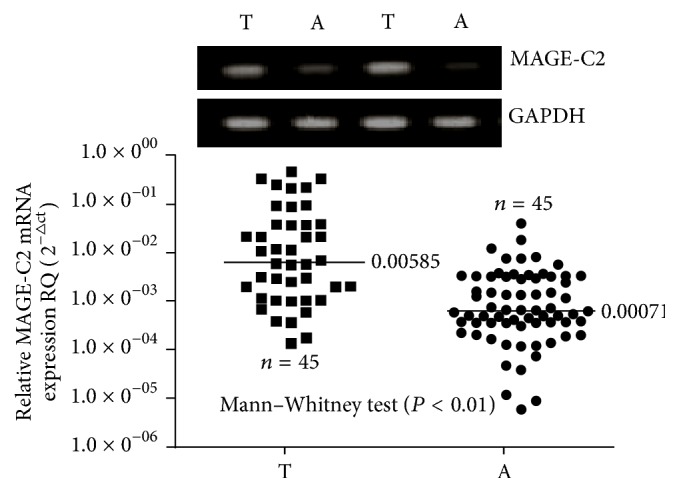
The MAGE-C2 protein was detected by RT-PCR. Note: A: adjacent tissues; T: triple-negative breast cancer tissues; RT-PCR: reverse transcription-polymerase chain reaction.

As the result of immunohistochemistry, the protein particles of MAGE-C2 mainly located in nucleus and cytoplasm of tumor and normal cells (Figures 1(a)–1(f)). According to the statistics, the high MAGE-C2 expression rate in TNBC tissues is 38.18% (42/110), and in adjacent tissues it is 9.09% (10/110); significant difference between the two exists (*P* < 0.05). In order to confirm the above result, 45 pairs of TNBC tissues and corresponding adjacent tissues were randomly selected by us to perform quantitative analysis. We analyzed the content of the MAGE-C2 protein in tumor and paracarcinoma tissues by western blotting. The results suggest that the content of MAGE-C2 protein in TNBC tissues is significantly higher than corresponding adjacent tissues (*P* < 0.05, Figure 2). The MAGE-C2 mRNA was measured to quantify RT-PCR which is also necessary. MAGE-C2 mRNA was detected in a higher level in TNBC tissues compared with adjacent tissues (*P* < 0.05); please see Figure 3. According to results of immunohistochemistry, all TNBC patients were divided into low MAGE-C2 expression group and high MAGE-C2 expression group. The MAGE-C2 mRNA levels of high MAGE-C2 expression group are significantly higher than low MAGE-C2 expression group (*P* < 0.001).

### 3.2. MAGE-C2 Expression and Clinicopathological Characteristics of TNBC Patients

In this study, 110 cases of TNBC patients were divided into low MAGE-C2 expression group and high MAGE-C2 expression group according to the previous criteria. Then, we investigated the relationship of MAGE-C2 expression status and clinicopathological parameters of TNBC patients.  Table 1 summarized the clinical and pathological findings of the patients with TNBC. The high MAGE-C2 expression levels in TNBC patients were closely associated with lymph node status, TNM stage, and lymphovascular invasion (*P* < 0.001). But, there was no relationship between MAGE-C2 expression levels and age, tumor size, and differentiation (*P* > 0.05).

### 3.3. MAGE-C2 Expression and Prognosis of TNBC Patients

**Figure 4:**
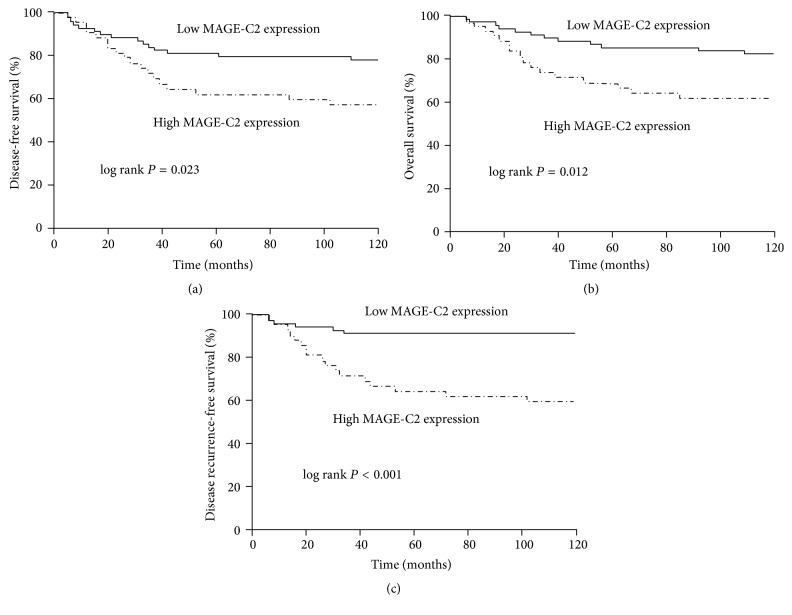
The MAGE-C2 expression and prognosis of patients with TNBC by Kaplan-Meier method based on immunohistochemistry. (a) DFS; (b) OS; (c) disease recurrence-free survival. Note: DFS: disease-free survival; OS: overall survival; TNBC: triple-negative breast cancer tissues.

In order to explore the relationship of MAGE-C2 status and prognosis of patient with TNBC, we plotted the survival curves (DFS and OS) by Kaplan-Meier method. The TNBC patients with high MAGE-C2 expression had significantly shorter survival time (DFS and OS) than low expression patients in accordance with the result of survival analysis (Figures 4(a) and 4(b)). Also noteworthy, that TNBC patients with high MAGE-C2 expression own higher risk of recurrence than TNBC patients with low MAGE-C2 expression (Figure 4(c)). The results of univariate and multivariate analysis were summarized in Tables 2 and 3. Univariate analysis showed that age, lymph node status, TNM stage, lymphovascular invasion, and MAGE-C2 expression status were closely associated with DFS and OS of patients with TNBC (*P* < 0.05). In the multivariate analysis, we found that age, lymph nodes, lymph node status, lymphovascular invasion of TNBC patients, and MAGE-C2 expression status were independent prognostic factors for DFS and OS (*P* < 0.05). It should be noted that high expression of MAGE-C2 maybe was an independent prognostic factor for DFS of TNBC patients (*P* = 0.041), but not entirely suitable for OS (*P* = 0.586). In addition, we carried out combined analysis of multiple risk factors, such as lymph node status plus MAGE-C2 expression, lymphovascular invasion plus MAGE-C2 expression, or lymph node status plus lymphovascular invasion. It is noteworthy that lymph node positive plus MAGE-C2 high expression implies poorer prognosis for TNBC patients (HR = 6.232, 95% CI = 3.069–13.115; *P* < 0.001).

## 4. Discussion

The members of the MAGE gene family possess the following common characteristics: being located on the X chromosome, being specifically expressed in multiple malignant tumors and normal testis and placenta, having an open reading frame, and containing a homologous sequence of about 200 amino acid residues in the C-terminus of the encoded protein [10–13]. There are 12 members in the family, and the most in-depth research has been performed on MAGE-A1 and MAGE-A3 [10, 13–16]. Recent results have indicated that the abnormal expression of MAGE-C2 is related to the genesis and development of multiple malignant tumors. However, there is no information on the relation between MAGE-C2 expression and TNBC. We adopted immunohistochemical methods in this study to evaluate the relations between MAGE-C2 expression and the clinical pathological features of patients with TNBC. We also applied RT-PCR and western blot techniques to further verify the results and found that MAGE-C2 showed high expression in TNBC tissues when compared with paracarcinoma tissues.

The possible reasons for the expression of MAGE genes in multiple malignant tumors are as follows [17–22]: (1) MAGE genes are related to the uncontrolled genetic regulation process in tumor tissues, (2) MAGE genes, which are heavily methylated in human somatic cells, are expressed, after demethylation, in malignant tumor cells, and (3) histones are deacetylated at the end of embryonic development, resulting in the inactivation of MAGE genes, which could be reactivated and abnormally expressed after a tumor is formed. We analyzed the relationships between the expression of MAGE-C2 and the clinical pathological features of TNBC patients and found that there were statistical differences between the MAGE-C2 expression level and the clinical staging, lymph node status, and lymphovascular invasion in TNBC patients, indicating that a high MAGE-C2 expression level may facilitate the invasion and metastasis of TNBC cells. However, the exact mechanism remains to be elucidated. We performed a survival analysis of the 110 TNBC patients through using the Kaplan-Meier method and Cox proportional hazard regression model, and the results indicated that a high MAGE-C2 expression level, which was the same as the clinical staging, was an independent parameter of a poor prognosis for TNBC patients: the higher the MAGE-C2 expression level was, the worse the prognosis for TNBC patients was.

In terms of tumor immunotherapy, MAGE-C2 may be a target for the immunotherapy. Therapy with autologous T cells that have been gene-engineered to express chimeric antigen receptors or T cell receptors provides a feasible and broadly applicable treatment for cancer patients. Previous clinical trials confirmed that this treatment is used to treat patients with MAGE-C2-positive tumors [23]. Expression of cancer testis antigens has been associated with prognosis in gastrointestinal stromal tumors and other malignancies. Cancer testis antigens are currently being investigated for cancer immunotherapy. CT10/MAGE-C2 and GAGE should be explored together with other previously described cancer testis antigens as targets for immunotherapy of gastrointestinal stromal tumors in cases [24]. Tumor cells exposed to interferon-gamma (IFN-*γ*) were better recognized by the anti-MAGE-C2(42–50) CTL clone. This mainly resulted from a better processing of the peptide by the immunoproteasome as compared to the standard proteasome. The patient was mediated by an antitumor response shaped by IFN-*γ* and dominated by CTL directed against peptides that are better produced by the immunoproteasome, such as the MAGE-C2 peptides [25]. A corollary was that purging cell cycle genes out of a signature failed to rule out the confounding effect of proliferation. Hence, it is questionable to suggest that a mechanism is relevant to human breast cancer from the finding that a gene expression marker for this mechanism predicts human breast cancer outcome [26]. Hence, multi-gene-signatures may be a better prediction; the alternative is to use the marker on several independent cohorts. For example, in Liu et al.'s study, they identify that pten/p53 tumors predicted poor survival for claudin-low patients [27]. This provides a new idea for us.

Therefore, we concluded that MAGE-C2 level could be an indicator of a poor TNBC prognosis. In addition, it could be a new target for TNBC immunotherapy. We believe that tumor infiltration and metastasis can be slowed by obstructing the relevant signal transduction pathway of MAGE-C2.

## Figures and Tables

**Table 1:** Associations between MAGE-C2 expression status and clinicopathologic parameters.

Variable	MAGE-C2		*P* value
	Low expression, *n* (%)	High expression, *n* (%)	
Total	68 (61.81)	42 (38.18)	—
Median age, years (range)	47 (22–79)	46 (27–67)	0.391
Age, *n* (%)			0.338
≤35	14 (20.59)	12 (28.57)	
>35	54 (79.41)	30 (71.43)	
Tumor size, *n* (%)			0.292
≤2 cm	19 (27.94)	8 (19.05)	
>2 cm	49 (72.06)	34 (80.95)	
Lymph node status, *n* (%)			<0.001
Negative	48 (70.59)	9 (21.43)	
Positive	20 (29.41)	33 (78.57)	
TNM stage, *n* (%)			<0.001
I/II	63 (92.65)	14 (33.33)	
III	5 (7.35)	28 (66.67)	
Differentiation, *n* (%)			0.792
Well	7 (10.29)	6 (14.29)	
Moderate/poor	61 (89.71)	36 (85.71)	
Lymphovascular invasion, *n* (%)			<0.001
Yes	4 (5.88)	21 (50.00)	
No	64 (94.12)	21 (50.00)	

TNM (tumor node metastasis) staging was conducted on all breast cancer patients postoperatively in accordance with the American Joint Committee on Cancer (AJCC, 7th edition). The Miller and Payne grading system was used for determining differentiation of the neoplasms.

**Table 2:** Univariate and multivariate analysis of disease-free survival in 110 patients.

Variables	Univariate			Multivariate		
	HR	95% CI	*P* value	HR	95% CI	*P* value
Age, years (≤35 versus >35)	0.408	0.202–0.821	0.012	0.367	0.174–0.773	0.008
Tumor size, cm (≤2 versus >2)	1.551	0.640–3.757	0.331	1.381	0.528–3.609	0.510
Lymph node status (negative versus positive)	5.096	2.207–11.768	<0.001	3.274	1.229–8.723	0.018
TNM stage (I/II versus II)	2.975	1.498–5.909	0.002	1.574	0.547–4.528	0.400
Differentiation (well versus moderate/poor)	4.346	0.594–31.816	0.148	2.720	0.325–22.744	0.356
Lymphovascular invasion (no versus yes)	4.490	2.257–8.931	<0.001	2.803	1.087–7.225	0.033
MAGE-C2 expression (low versus high)	2.170	1.092–4.311	0.027	1.513	1.168–3.567	0.041

**Table 3:** Univariate and multivariate analysis of overall survival in 110 patients.

Variables	Univariate			Multivariate		
	HR	95% CI	*P* value	HR	95% CI	*P* value
Age, years (≤35 versus >35)	0.366	0.173–0.775	0.009	0.353	0.160–0.779	0.010
Tumor size, cm (≤2 versus >2)	1.565	0.595–4.118	0.364	1.392	0.491–3.945	0.534
Lymph node status (negative versus positive)	4.946	2.002–12.219	0.001	2.733	1.947–7.887	0.043
TNM stage (I/II versus III)	3.287	1.558–6.935	0.002	1.476	0.468–4.654	0.507
Differentiation (well versus moderate/poor)	3.595	0.488–26.465	0.209	2.413	0.280–20.766	0.423
Lymphovascular invasion (no versus yes)	4.875	2.310–10.287	<0.001	2.730	1.964–7.725	0.049
MAGE-C2 expression (low versus high)	2.517	1.189–5.329	0.016	0.726	0.229–2.299	0.586
